# RISK FACTORS FOR DISABLEMENT WITH AGING IN CENTRAL MEXICO

**DOI:** 10.1093/geroni/igad104.0682

**Published:** 2023-12-21

**Authors:** Lucia Caudillo-Ortega, Shelley Blozis, Nancy Blanco-Arroyo, Tracie Harrison

**Affiliations:** Universidad de Guanajuato, Guanajuato, Guanajuato, Mexico; UC Davis, Davis, California, United States; The University of Texas at Autin, Austin, Texas, United States; The University of Arkansas for Medical Sciences, Little Rock, Arkansas, United States

## Abstract

Health, function, and social participation (e.g., disablement) were studied among community living men and women with mobility limitations living in central Mexico. Psychological (i.e., depression and activity effort), cultural (i.e., attitudes toward assistive devices) and social (i.e., personal resources) variables were tested with the goal to identify differential risk for worse health (Charlson Comorbidity Index) outcomes. We hypothesized significant differences based on psychological, social, and cultural influences. Data was collected using in-person, Spanish language surveys from 2019-2020. Measures were reliable and valid. The sample (N=257) were an average of 70.98 years (std 7.72) with an education of 6.97 years (std 7.22) and onset of functional limitation at 62.5 (std 15.07). Most reported female sex (51%). Using machine learning regression technique, statistically significant interactions were found: (1) Age was an important predictor of health; individuals clustered according to different age groups; those below 65 years had the best health; followed by those between 65 and 70 years, followed next by those between 70 and 75 years, and those over 75 years having the worst health, on average. (2) Age, biological sex, and depression were important predictors of function. Further, for those below 83 years, depression was related to function; specifically, for these individuals, those with depression levels above 18.3 had worse functioning relative to those who depression score was below 18.3. (3) age was an important predictor of social participation. For those with mobility limitations, age, sex, and depression were risks factors for disablement in Mexico.

